# Clinical Insights to Complete and Incomplete Surgical Revascularization in Atrial Fibrillation and Multivessel Coronary Disease

**DOI:** 10.3389/fcvm.2022.910811

**Published:** 2022-06-15

**Authors:** Michal Pasierski, Jakub Staromłyński, Janina Finke, Radoslaw Litwinowicz, Grzegorz Filip, Adam Kowalówka, Wojciech Wańha, Michalina Kołodziejczak, Natalia Piekuś-Słomka, Andrzej Łoś, Sebastian Stefaniak, Wojciech Wojakowski, Marek Jemielity, Jan Rogowski, Marek Deja, Dariusz Jagielak, Krzysztof Bartus, Silvia Mariani, Tong Li, Matteo Matteucci, Daniele Ronco, Federica Jiritano, Dario Fina, Gennaro Martucci, Paolo Meani, Giuseppe Maria Raffa, Artur Słomka, Pietro Giorgio Malvidni, Roberto Lorusso, Michal Zembala, Piotr Suwalski, Mariusz Kowalewski

**Affiliations:** ^1^Department of Cardiac Surgery, Central Clinical Hospital of the Ministry of Interior, Centre of Postgraduate Medical Education, Warsaw, Poland; ^2^Thoracic Research Centre, Innovative Medical Forum, Collegium Medicum Nicolaus Copernicus University, Bydgoszcz, Poland; ^3^Department of Cardiovascular Surgery and Transplantology, Jagiellonian University Medical College, John Paul II Hospital, Kraków, Poland; ^4^Department of Cardiac Surgery, School of Medicine in Katowice, Medical University of Silesia, Katowice, Poland; ^5^Department of Cardiac Surgery, Upper-Silesian Heart Center, Katowice, Poland; ^6^Department of Cardiology and Structural Heart Diseases, Medical University of Silesia, Katowice, Poland; ^7^Department of Anaesthesiology and Intensive Care, Antoni Jurasz University Hospital No. 1, Collegium Medicum Nicolaus Copernicus University, Bydgoszcz, Poland; ^8^Division of Cardiology, Yale School of Medicine, New Haven, CT, United States; ^9^Department of Inorganic and Analytical Chemistry, Ludwik Rydygier Collegium Medicum in Bydgoszcz, Nicolaus Copernicus University, Toruń, Poland; ^10^Department of Cardiac and Vascular Surgery, Medical University of Gdańsk, Gdańsk, Poland; ^11^Department of Cardiac Surgery and Transplantology, Poznań University of Medical Sciences, Poznań, Poland; ^12^Cardio-Thoracic Surgery Department, Heart and Vascular Centre, Maastricht University Medical Centre, Cardiovascular Research Institute Maastricht, Maastricht, Netherlands; ^13^Department of Cardiac Surgery, University Hospital Düesseldorf, Düesseldorf, Germany; ^14^Department of Cardiac Surgery, Circolo Hospital, University of Insubria, Varese, Italy; ^15^Department of Cardiac Surgery, University Magna Græcia of Catanzaro, Catanzaro, Italy; ^16^Department of Cardiothoracic and Vascular Anesthesia and Intensive Care Unit (ICU), Istituto di Ricovero e Cura a Carattere Scientifico (IRCCS) Policlinico San Donato, Milan, Italy; ^17^Anesthesia and Intensive Care Department, Istituto di Ricovero e Cura a Carattere Scientifico (IRCCS)-Istituto Mediterraneo per i Trapianti e Terapie ad Alta Specializzazione (ISMETT), Palermo, Italy; ^18^Cardiac Surgery Unit, Istituto di Ricovero e Cura a Carattere Scientifico (IRCCS)-Istituto Mediterraneo per i Trapianti e Terapie ad Alta Specializzazione (ISMETT), Palermo, Italy; ^19^Department of Pathophysiology, Ludwik Rydygier Collegium Medicum Bydgoszcz, Nicolaus Copernicus University, Toruń, Poland; ^20^Cardiac Surgery Unit, Lancisi Cardiovascular Center, Polytechnic University of Marche, Ancona, Italy; ^21^Department of Cardiac, Vascular and Endovascular Surgery and Transplantology, Silesian Center for Heart Diseases, Zabrze, Poland

**Keywords:** atrial fibrillation, CABG, complete revascularization, survival, long-term

## Abstract

**Objectives:**

Although endorsed by international guidelines, complete revascularization (CR) with Coronary Artery Bypass Grafting (CABG) remains underused. In higher-risk patients such as those with pre-operative atrial fibrillation (AF), the effects of CR are not well studied.

**Methods:**

We analyzed patients’ data from the HEIST (HEart surgery In AF and Supraventricular Tachycardia) registry. Between 2012 and 2020 we identified 4770 patients with pre-operative AF and multivessel coronary artery disease who underwent isolated CABG. We divided the cohort according to the completeness of the revascularization and used propensity score matching (PSM) to minimize differences between baseline characteristics. The primary endpoint was all-cause mortality.

**Results:**

Median follow-up was 4.7 years [interquartile range (IQR) 2.3–6.9]. PSM resulted in 1,009 pairs of complete and incomplete revascularization. Number of distal anastomoses varied, accounting for 3.0 + –0.6 vs. 1.7 + –0.6, respectively. Although early (< 24 h) and 30-day post-operative mortalities were not statistically different between non-CR and CR patients [Odds Ratio (OR) and 95% Confidence Intervals (CIs): 1.34 (0.46–3.86); *P* = 0.593, Hazard Ratio (HR) and 95% CIs: 0.88 (0.59–1.32); *P* = 0.542, respectively] the long term mortality was nearly 20% lower in the CR cohort [HR (95% CIs) 0.83 (0.71–0.96); *P* = 0.011]. This benefit was sustained throughout subgroup analyses, yet most accentuated in low-risk patients (younger i.e., < 70 year old, with a EuroSCORE II < 2%, non-diabetic) and when off-pump CABG was performed.

**Conclusion:**

Complete revascularization in patients with pre-operative AF is safe and associated with improved survival. Particular survival benefit with CR was observed in low-risk patients undergoing off-pump CABG.

## Introduction

Although never compared directly in a randomized controlled trial (RCT), complete revascularization (CR) during coronary artery bypass grafting (CABG) is considered to be superior to incomplete revascularization (ICR) in multi-vessel coronary artery disease (MV-CAD). The benefit is thought to originate from reduced risk of future cardiovascular events, namely periprocedural myocardial infarction (MI) and repeat revascularization (RR). Many observational studies, as well as insights from subgroup analysis of RCTs reinforced this notion (1–3). Several RCTs which investigated CR in the context of percutaneous intervention (PCI) for ST-elevation myocardial infarction (STEMI) have shown the benefit of complete, compared to ICR for MV-CAD (4–6). However, surgical revascularization is still the first-choice procedure in high-risk non-acute MI patients, specifically those with diabetes (7) and intermediate-to-high anatomical complexity coronary disease (8).

The 2018 ESC/EACTS guidelines on myocardial revascularization emphasized that the expected highest completeness of revascularization should guide the choice of treatment strategy (9). The question arises if surgeons should attempt CR at all costs and, if not, what type of risk factors may discourage them from pursuing one. Atrial fibrillation (AF) is an independent predictor of mortality and morbidity after CABG (10, 11). Apart from an increased risk of stroke, AF is also associated with an over a fourfold increased risk of developing heart failure (12). Moreover, an impaired graft flow in AF CABG patients was observed (13). Most reports estimate the pre-op AF prevalence in CABG patients at 6–10%, but in some reports, it was as high as 20% (14). Because of aging of the society, the prevalence of AF is likely to rise.

The disparity in reported results of CR in CABG, and the shortage of evidence in high-risk patients, requires further investigation (15, 16). The current study aimed to address whether there exists a survival benefit with CR in MV-CAD and underlying AF.

## Patients and Methods

### Study Population and Clinical Variables

Because of the retrospective nature of the study, the ethics committee approval was waived. Our investigation was a part of the HEIST (Heart Surgery In atrial fibrillation and Supraventricular tachycardia) Registry (NCT04860882). We included consecutive AF patients, over 18 year old, admitted to 8 tertiary centers in Poland, Netherlands, and Italy between January 2012 and December 2020 who had isolated CABG for MV-CAD performed (Supplementary Figure 1). Patients who (1) had no diagnosis of AF; (2) had CABG with concomitant valvular or aortic procedures, were not included in the study. Similarly, (3) patients with single-vessel CAD or (4) patients in whom the number of distal anastomoses and/or type of graft material used could not be determined were excluded from the analyses. (5) Patients undergoing hybrid revascularization by intention-to-treat protocol, or who were admitted for (6) staged revascularization strategy or (7) re-do surgery were not included.

### Endpoints and Definitions

The primary endpoint was all-cause mortality following complete vs. incomplete surgical revascularization for MV-CAD. We defined CR as grafting two significantly stenotic lesions in two-vessel disease and three lesions in three-vessel disease of different territories: right coronary artery (RCA), left anterior descending- (LAD), and circumflex- (Cx) artery. Additional grafts to the diseased systems were encouraged and when the number of grafts was greater than the number required for CR, the approach was considered as “supracomplete” revascularization (SCR). Only the coronary vessels with significant stenosis were bypassed. We defined ICR as failure to graft two significantly stenotic lesions in two-vessel disease and three lesions in three-vessel disease of different territories for whatever reason (15). Each distal anastomosis was counted as a separate graft, e.g., sequential conduit was counted as more than one graft. Whenever the territory that sequential graft supplied couldn’t be determined from the registry or this data was missing, it was not taken into consideration when assessing the completeness of revascularization. We report data on early post-operative (< 24 h) mortality rates, in-hospital complications, lengths of stay in the intensive care unit (ICU) and in the hospital (HLoS).

### Statistical Analyses

Continuous variables were summarized as mean with standard deviation if normally distributed; non-normal distributions were summarized as median with IQR and compared with the Mann–Whitney U test or standard *t*-test, as appropriate. Categorical variables [number (%)] were compared with the Fisher exact test. Propensity matching was generated for each patient from a non-parsimonious multivariable logistic regression model that was based on baseline characteristics (age, number of vessels diseased, comorbidities, EuroSCORE II, LVEF, CCS, NYHA, and others listed in Table 1) and procedural [concomitant ablation, type of surgery (Off-Pump, On-Pump), procedure urgency] covariates as independent variables with treatment type (CR vs. non-CR) as a dependent variable. We used and opt-match and matchIt packages, 1-to-1 pairing, without replacement within a specific caliper width of 0.2 standard deviation of the propensity score. We computed standardized mean differences (SMDs) to verify the balance between CR versus non-CR groups after matching (Supplementary Figure 2). Risk Ratios (RRs) were used for in-hospital outcomes, whereas Cox proportional-hazards models were used to determine factors related to the event-free survival at long-term follow-up. We calculated Hazard Ratios (HRs) point estimate and 95% confidence intervals (95% CIs) with ensuing statistical models. Mortality was assessed with Kaplan–Meier survival curves fitted after PS matching. As a further sensitivity analysis, defined subgroup analyses were performed to assess the mortality in different scenarios. STATA MP v13.0 software (StataCorp, College Station, TX, United States) and R (with Rcmdr package and EZR software) were used for computations.

**TABLE 1:** Pre-operative characteristics after PS-matching.

	Total (2018)	Non-CR (1009)	CR (1009)	*P*-value
Baseline characteristics				
Age years [median (IQR)]	70 (64–76)	70 (64–76)	70 (64–76)	0.792
Male gender	1589 (78.7)	795 (78.8)	794 (78.7)	0.99
EuroSCORE II	1.91 (1.21,3.19)	1.92 (1.22, 3.19)	1.90 (1.18, 3.19)	0.760
Diabetes	899 (44.5)	457 (45.3)	442 (43.8)	0.531
Insulin ± oral hypoglycemic drugs	372 (18.4)	186 (18.4)	186 (18.4)	0.99
Smoking	1247 (61.8)	628 (62.2)	619 (61.3)	0.714
Hypertension	1829 (90.6)	917 (90.9)	912 (90.4)	0.760
Hyperlipidemia	1293 (64.1)	656 (65.0)	637 (63.1)	0.404
BMI [median (IQR)]	28.69 (25.78–31.70)	28.70 (25.65, 31.71)	28.69 (25.95, 31.67)	0.815
Pulmonary hypertension^a^	154 (7.6)	86 (8.5)	68 (6.7)	0.154
Renal impairment	1128 (55.9)	570 (56.5)	558 (55.3)	0.622
Dialysis (regardless of CC)	26 (1.3)	13 (1.3)	13 (1.3)	0.99
Peripheral artery disease	515 (25.5)	263 (26.1)	252 (25.0)	0.610
Cerebrovascular disease	162 (8)	75 (7.4)	87 (8.6)	0.368
History of stroke	73 (3.6)	34 (3.4)	39 (3.9)	0.634
History of TIA/RIND	72 (3.6)	28 (2.8)	44 (4.4)	0.071
chronic lung disease	111 (5.5)	56 (5.6)	55 (5.5)	0.99
LVEF (%) [median (IQR)]^a^	50 (40–55)	50 (40–55)	49 (40–55)	0.527
3 vessel CAD	1609 (79.7)	813 (80.6)	796 (78.9)	0.376
Previous MI	1076 (53.3)	517 (51.2)	559 (55.4)	0.067
Previous PCI	304 (15.1)	152 (15.1)	152 (15.1)	0.99
NYHA IV	31 (1.5)	14 (1.4)	17 (1.7)	0.718
CCS 4	194 (9.6)	93 (9.2)	101 (10.0)	0.597
ACS	95 (4.7)	47 (4.7)	48 (4.8)	0.99

*^a^Missing data.*

*PS, propensity score; IQR, interquartile range; BMI, body mass index; PA, pulmonary artery; CC, creatinine clearance; TIA, transient ischemic attack; LVEF, left ventricle ejection fraction; CAD, coronary artery disease; VD, vessel disease; MI, myocardial infarction; PCI, percutaneous coronary intervention; NYHA, New York Heart Association; CCS, Canadian Cardiovascular Society; ACS, Acute Coronary Syndrome.*

## Results

**FIGURE 1:**
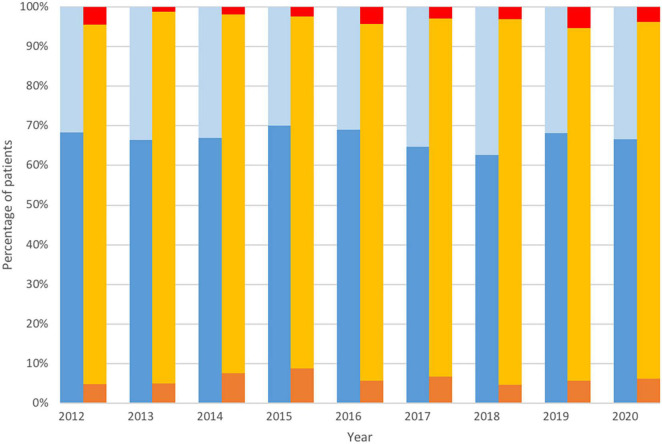
Temporal trends in completeness of revascularization and utilization of arterial grafting. Complete revascularization (dark blue), non-complete revascularization (light blue), multiple arterial grafting (orange), single arterial grafting (yellow), total arterial revascularization (red).

We identified 4,770 patients with pre-operative AF undergoing CABG; of those, in 3,193 (66.9%) patients, CR according to predefined criteria was achieved. During the 9-year follow-up, there were no marked differences in the proportion of complete vs. ICR, nor there were any differences in the adoption of multi-arterial grafting (MAG) (Figure 1). Using the propensity score matching (PSM) model, two groups of 1,009 patients each were determined, by pairing non-CR patients with CR controls to achieve similar baseline (Table 1) and surgical (Table 2) characteristics. We report details on matching quality in Supplementary Figure 2.

**TABLE 2:** Operative characteristics after PS-matching.

	Total (2018)	Non-CR (1009)	CR (1009)	*P*-value
Procedural characteristics				
Critical pre-operative state	43 (2.1)	22 (2.2)	21 (2.1)	0.99
IABP	8 (0.4)	4 (0.4)	4 (0.4)	0.99
iv. inotropes	35 (1.7)	19 (1.9)	16 (1.6)	0.734
Mechanical vent	8 (0.4)	5 (0.5)	3 (0.3)	0.726
Emergency surgery	74 (3.7)	37 (3.7)	37 (3.7)	0.99
OPCAB	1031 (51.1)	531 (52.6)	500 (49.6)	0.182
CPB (min)^a^	74 (55–95)	60 (45–74)	90 (71–109)	< 0.001
X-clamp (min)^a^	41 (29–54)	32 (24–41)	50 (40–61)	< 0.001
Conversion to ONCAB	17 (0.8%)	11 (1.1)	6 (0.6)	0.330
Concomitant ablation	79 (3.9)	41 (4.1)	38 (3.8)	0.819

*^a^Missing data.*

*PS, propensity score; CPR, cardiopulmonary resuscitation; IABP, intra-aortic balloon pump; iv, intravenous; OPCAB, Off-Pump Coronary Artery Bypass; ONCAB, On-Pump Coronary Artery Bypass; CPB, cardiopulmonary bypass; LAAO, left atrial appendage occlusion; ±; SD, Standard Deviation.*

### Baseline and Surgical Characteristics

Baseline characteristics were balanced between groups, with similar EUROSCORE values [median (IQR): 1.92 (1.22–3.19) and 1.90 (1.18–3.19), respectively in patients with incomplete and CR]. The median age was identical in both groups – 70 years (64–76). Of included patients, 78.8% (non-CR) vs. 78.7% (CR) were men, 80.6% of non-CR patients in comparison with 78.9% in the CR group had a 3-vessel coronary artery disease (*p* = 0.376). Concomitant ablation was performed in 4.1% of non-CR and 3.8% of the CR group; 531 (52.6%) non-CR and 500 (49.6%) CR patients were operated on without the use of cardio-pulmonary bypass (*p* = 0.182). As expected, the number of distal anastomoses varied between groups (1.7 ± 0.6 non-CR vs. 3.0 ± 0.6 CR, *P* < 0.001). Table 3 lists information regarding grafts and anastomoses.

**TABLE 3:** Grafts and anastomoses after PS-matching.

	Total (2018)	Non-CR (1009)	CR (1009)
LIMA	1860 (92.2)	914 (90.5)	950 (94.2)
RIMA	83 (4.1)	35 (3.5)	48 (4.8)
BIMA	77 (3.8)	34 (3.4)	43 (4.3)
Pedicled IMA^a^	619 (30.7)	295 (34.6)	324 (38.0)
Skeletonized IMA^a^	1085 (53.8)	557 (65.4)	528 (62.0)
Radial artery	44 (2.2)	25 (2.5)	19 (1.9)
Multiple arterial grafts	203 (10.1)	82 (8.1)	121 (12.0)
Total Arterial Revascularization	42 (2.1)	0 (0.0)	42 (4.2)
Number of anastomoses (Mean + –SD)		2 (1–2)	3 (3–3)

*^a^Missing data.*

*LIMA/RIMA/BIMA, Left/Right/Bilateral Internal Mammary Artery; RA, Radial Artery. ±; SD, Standard Deviation.*

### Clinical Outcomes

**FIGURE 2:**
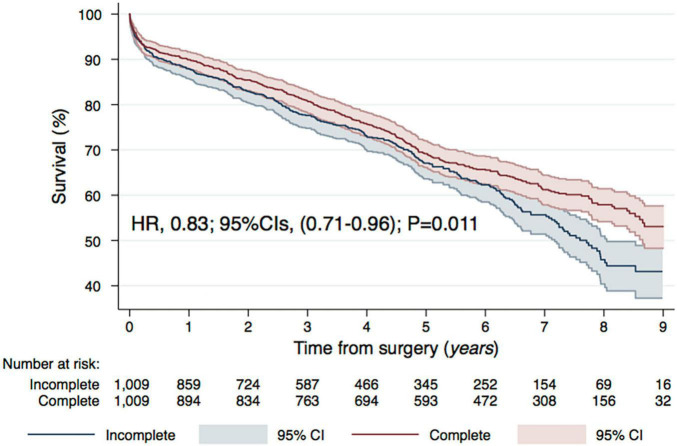
Long-term mortality, propensity matched Kaplan–Meier survival curves between the two groups: CR vs. non-CR CABG for the analysis of long-term survival. Hazard Ratios and respective 95% Confidence Intervals.

In-hospital outcomes and post-operative complications were consistent between groups (Table 4). Early mortality (24 h) and 30-day mortality were unaffected by CR [Odds Ratio (OR) and 95% Confidence Intervals (CIs): 1.34 (0.46–3.86), *P* = 0.593, Hazard Ratio (HR) and 95% CIs: 0.88 (0.59–1.32), *P* = 0.542, respectively]. Cardiac tamponade and/or re-thoracotomy for bleeding occurred in 3.1 vs. 5.1% and was statistically more frequent in the CR group [Risk Ratio (RR) and 95% CIs, 1.65 (1.06–2.55), *P* = 0.032]. Cardiopulmonary bypass (CBP) and aortic X-clamp times were significantly longer in the CR group: the median of CBP time was 65 vs. 79 min (*P* ≤ 0.001) in the CR and non-CR group and respectively 34 vs. 40 min (*P* ≤ 0.001) of aortic X-clamp time. In the long-term follow-up [As stated in the abstract median follow up was 4.7 years (2.3–6.9)], CR was associated with significantly lower mortality [HR (95% CIs) 0.83 (0.71–0.96), *P* = 0.011] (Figure 2).

**TABLE 4:** In-hospital outcomes after PS-matching.

	Non-CR (1009)	CR (1009)	Risk ratio (95% CIs)	*P*-value
Early post-operative mortality (< 24 h)	7 (0.7)	8 (0.8)	1.14 (0.42–3.14)	0.288
Cardiac tamponade and/or rethoracotomy for bleeding	31 (3.1)	51 (5.1)	1.65 (1.06–2.55)	0.032
Respiratory failure	60 (5.9)	74 (7.3)	1.23 (0.89–1.71)	0.245
Neurologic complications	25 (2.5)	21 (2.1)	0.84 (0.47–1.49)	0.655
Multiorgan failure	21 (2.1)	21 (2.1)	1.00 (0.55–1.82)	1.000
Gastrointestinal complications	13 (1.3)	16 (1.6)	1.23 (0.6–2.55)	0.709
Acute kidney failure and/or dialysis	32 (3.2)	34 (3.4)	1.06 (0.66–1.71)	0.901
Superficial sternal wound infection	19 (1.9)	21 (2.1)	1.11 (0.60–2.04)	0.873
Deep sternal wound infection	18 (1.8)	14 (1.4)	0.78 (0.39–1.56)	0.594
Mediastinitis	4 (0.4)	6 (0.6)	1.50 (0.42–5.3)	0.753
PPI	4 (0.4)	4 (0.4)	1.00 (0.25–3.99)	1.000
ECMO	1 (0%)	1 (0%)	1.00 (0.06–15.97)	1.000
IABP	18 (1.8%)	21 (2.1%)	1.17 (0.63–2.18)	0.628

*PS, propensity score; CIs, confidence intervals; MI, myocardial infarction; ICU, intensive care unit; PPI, permanent pacemaker implantation; ECMO, extracorporeal membrane oxygenation; IABP, intra-aortic balloon pump.*

**FIGURE 3:**
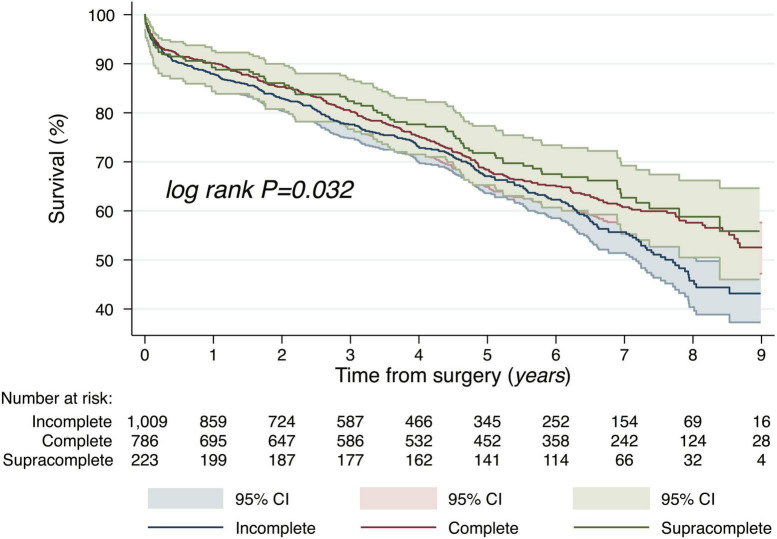
Long-term mortality, propensity matched Kaplan–Meier survival curves between three groups: CR, non-CR, SCR CABG. Logrank test for differences in survival between groups. CR, complete revascularization; SCR, “supracomplete” revascularization.

The CR group was further divided into patients who underwent complete- and “supracomplete” revascularization. The latter was associated with an even greater reduction in mortality HR (95% CIs) 0.76 (0.59–0.97), *P* = 0.023 (for SCR vs. ICR). Between ICR, CR, and SCR we observed a significant trend toward lower mortality (log rank *P* = 0.032, Figure 3).

**FIGURE 4:**
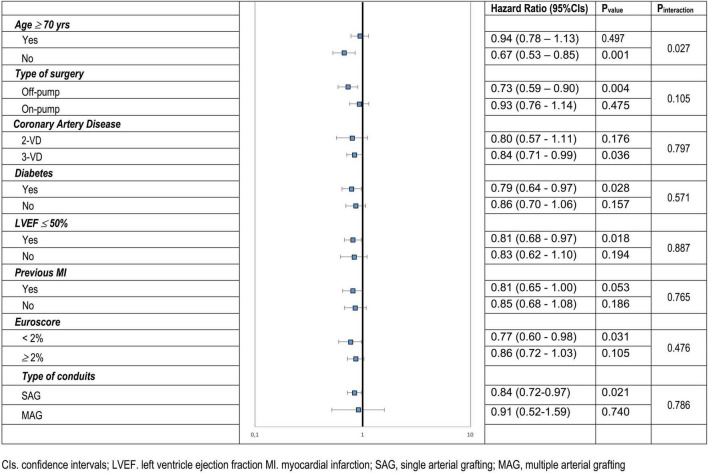
Subgroup Analysis, Hazard Ratios and 95% Confidence Intervals (Cis) for death from any cause in CR as compared to non-CR according to selected characteristics; CR, complete revascularization; VD, vessel disease; LVEF, left ventricle ejection fraction; MI, myocardial infarction.

In subgroup analyses, the benefit of improved long-term survival was sustained across diverse patient populations. Especially beneficent were younger (< 70 years old.) patients [HR (95% CIs) 0.67 (0.53–0.85), *P* = 0.001 for < 70 year old Vs. HR (95% CIs) 0.94 (0.78–1.13), *P* = 0.497 for ≥ 70 year old; *P* interaction = 0.027]. The effect was also more pronounced in patients with lower EuroSCORE II, without diabetes and when off-pump CABG was performed. Further details on the subgroup analyses are shown in Figure 4.

## Discussion

The current analysis is the first to focus on the long-term results of complete and incomplete surgical revascularization for MV-CAD in patients with pre-existing AF. Its main findings are as follows; (1) there was a high rate of ICR; (2) long-term benefit of CR; (3) even greater benefit with a higher number of additional grafts; (4) low prevalence of MAG and TAR in the population of AF patients, without significant temporal trends.

Complete revascularization, especially achieved through CABG, is characterized by improved long-term survival and a lower rate of reinterventions compared with ICR (1–3). It remains to be established whether this distinction is specifically because of ICR as a surgical method, deficiency, or anatomical obstacles during CABG or, whether the ICR is only a marker of more advanced and progressive coronary disease. The ICR usually indicates complex coronary pathology, with unfavorable outcomes originating from the patient’s baseline risk profile. In reality, even though ICR may contrarily influence long-term results (17), it may be the most appropriate treatment method in a specific subset of prohibitive-risk patients. When the risks of surgery must be minimized to reduce perioperative mortality and complications, target vessel revascularization represents possibly the best feasible course of action (6).

Patients presenting with AF are at markedly elevated, yet non-prohibitive, operative risk, nor is AF itself accounted for in prognostic scores (e.g., EuroSCORE II). Although, the prevalence of AF in patients undergoing CABG is much lower than in patients undergoing mitral valve surgery (18, 19), up to 20% of patients presenting for coronary surgical procedures may have preoperative AF (14, 20), which is often used as a marker for high-risk patients (10, 11, 21). This percentage rises with age and decreased left ventricular function, which is seen in an increasing number of patients referred for CABG surgery. Although no data exists on performing CR in this population, because of their high-risk nature, surgeons may be reluctant to aim for CR as it is associated with longer operative time. Until now, no single study has focused on a comparison of CR/ICR in the AF population undergoing CABG.

One subgroup analysis of the Atrial Fibrillation undergoing Coronary Artery Stenting (AFCAS) registry focusing on the impact of ICR, has shown that of 445 (46.8%) PCI subjects in whom physicians opted for ICR, at 1-year follow-up, had a higher rate of the composite endpoint of acute MI, stent thrombosis and RR, compared to patients with CR (13.9% vs. 9.4%, *p* = 0.003) (22). In an adjusted multivariable analysis, only creatinine clearance (inverse relationship) and ICR were independently associated with a higher risk of the composite endpoint [HR (95% CIs) 1.66, (1.10–2.50), *p* = 0.013] (22).

The latest reports and registries analyses present data on safety and efficacy of surgical CR in AF. In a study of 900 patients with end-stage renal disease, where 14.1% of all patients had pre-existing AF, emergency surgery, diabetes mellitus, the number of vein grafts and age were identified as risk factors for mortality (23). CR, the use of an internal thoracic artery and the sinus rhythm pre-op were recognized as beneficial factors for long-term survival (23). Although AF was not identified as an independent risk factor for perioperative mortality (*p* = 0.59), it was an independent predictor for late mortality (*p* < 0.001) (23). In an analysis of the KROK registry Off-Pump CABG offered a 30-day survival benefit to patients undergoing CABG surgery and presenting with underlying AF (24). On-Pump CABG, on the other hand, was associated with significantly improved long-term survival. CR was possible in 67.5% of patients and was significantly higher, by 10%, in patients undergoing On-Pump CABG (73.3 vs. 62.6%; *P* < 0.001) (24).

One finding of the preset report requires special attention; it was demonstrated that “supra-complete” revascularization, may further improve survival in AF patients undergoing CABG. A study by Schwann et al. investigated the effects of SCR in SAG and MAG and concluded that it conveyed a survival benefit in patients with 3-VD in a single arterial grafting group (which is the majority in our study) (25). Conversely, Chu et al. observed no survival benefit with multiple grafts to each myocardial territory (26). Supra-complete revascularization could be beneficial in several ways, by securing the vulnerable myocardium during the early phase post-op, particularly prone to arrhythmias and disturbances in blood flow, or protecting distal coronary arteries from MI in long-term when functionally non-significant and non-revascularized lesions become significant (27). However, aiming for SCR must increase operative times and since its benefit is not well established the surgeons face a difficult decision. Our results suggest that preoperative AF, although a poor prognostic factor in general, should not be deemed prohibitive while considering additional grafts to each coronary territory.

Several factors, beyond the completeness of the revascularization, can influence the outcome in the AF population. Analysis of the KROK registry (28) showed a significant survival benefit associated with concomitant surgical ablation (SA) in the setting of CABG. The same analysis showed that it remains severely underused, as it was performed only in 4.4%. Recent guidelines give a recommendation to concomitant SA during CABG surgery. Considering that both CR and SA prolong operative times, surgeons might decide to choose SA over the additional graft that would ensure completeness of the revascularization. Our results suggest that in non-prohibitive risk patients, both SA and CR should be aimed for. Another analysis of the KROK registry showed that patients undergoing multiple arterial grafting have survival benefits at long-term follow-up (13 years post-op) as compared to single arterial grafting (29). This benefit was further sustained in subgroup analyses, yet most appraised in low risk patients (< 70-year-old; EuroSCORE < 2; no diabetes) and when CR was achieved (*P* = 0.009). Some studies suggest that benefit with the CR may be conferred to SAG patients, wheares when multiple arterial grafts are used their superior patency could neutralize survival benefit associated with completeness of revascularization (30). Indeed, in our sensitivity analysis the benefit in MAG group was non-significant, although it has to be noted that the number of MAG patients was low.

## Limitations

Our study has several limitations. First, only all-cause mortality assessment is possible; the information regarding the cause of death, reinterventions, MIs, heart failure hospitalizations, adherence to anticoagulation therapy, or angiographic patency follow-up is not recorded in the registry. Second, although we addressed a potential selection bias, with propensity score matching according to baseline clinical variables, several confounders could prevail, an important of which is the lack of coronary angiograms that would allow us to access the percentage of chronic total occlusions. Additionally, we did not include the grafting choice (arterial vs. venous grafting) and patient allocation in the propensity score model. Third, detailed anatomy of coronary vessels is not available and therefore the feasibility of CR in each case could not be assessed. Finally, our data regarding coronary revascularization concerns CABG surgery only. Perhaps, some patients, in whom CR during surgery was deemed infeasible, could benefit from a staged hybrid revascularization with PCI as a second stage. Unfortunately, the registry at that time did not gather data regarding subsequent interventions.

## Conclusion

In this multicenter retrospective propensity-matched study of patients with preoperative AF, CR during CABG was associated with improved long-term survival. The particular benefit was observed in lower-risk patients. A significant trend was observed toward lower mortality with “supracomplete” revascularization.

## Data Availability Statement

The original contributions presented in the study are included in the article/Supplementary Material, further inquiries can be directed to the corresponding author.

## Ethics Statement

Ethical review and approval was not required for the study on human participants in accordance with the local legislation and institutional requirements. Written informed consent for participation was not required for this study in accordance with the national legislation and the institutional requirements.

## Author Contributions

MP, JS, JF, and MKow: conception and design. PS, RLo, MD, WWo, JR, MJ, MZ, and KB: administrative support. JS, RLi, GF, AK, WWa, AŁ, SS, DJ, FJ, MM, GM, and GR: provision of study materials or patients. NP-S, SM, TL, DR, and AS: collection and assembly of data. MP, MKow, MKoł, PGM, PM, and DF: data analysis and interpretation. MP, JS, JF, MKow, MKoł, and SM: manuscript writing. All authors approved the final manuscript.

## Conflict of Interest

The authors declare that the research was conducted in the absence of any commercial or financial relationships that could be construed as a potential conflict of interest.

## Publisher’s Note

All claims expressed in this article are solely those of the authors and do not necessarily represent those of their affiliated organizations, or those of the publisher, the editors and the reviewers. Any product that may be evaluated in this article, or claim that may be made by its manufacturer, is not guaranteed or endorsed by the publisher.
