# Supplementary material for: Clinical Insights to Complete and Incomplete Surgical Revascularization in Atrial Fibrillation and Multivessel Coronary Disease
Source: Front Cardiovasc Med. 2022 Jun 15;9:910811. doi: 10.3389/fcvm.2022.910811 (PMC9240216; doi:10.3389/fcvm.2022.910811)
Supplement: Supplementary file 1 [file Data_Sheet_1.docx]

**Clinical Insights to complete and incomplete surgical revascularization in atrial fibrillation and multivessel coronary disease. Analysis from the HEIST Registry.**

**SUPPLEMENTARY MATERIAL**

Supplementary Figure 1. Study flow-chart

Supplementary Figure 2. SMDs between CR and non-CR before and after PS matching

**Supplementary Figure 1.**

**Retrospective review 2012-2020**

Coronary artery bypass grafting surgery (N = 85,771)

Exclusion criteria

- baseline heart rhythm not AF or not reported

- single vessel CAD

- concomitant procedures

- number of distal anastomoses and/or type of grafts could not be determined

- <18 y.o.

-Patients undergoing hybrid revascularization, or

-Staged revascularization strategy or

-Re-do surgery

Coronary artery bypass grafting surgery for MV CAD and AF (N = 4,770)

Complete Revascularization

(N = 3,193)

Non Complete Revascularization

(N = 1,577)

PS matching

Complete Revascularization

(N = 1,009)

Non Complete Revascularization

(N = 1,009)

**Supplementary Figure 2. Standardized Mean Differences before and after PS matching**
